# Supplementary material for: Nanoscale characterization of the biomolecular corona by cryo-electron microscopy, cryo-electron tomography, and image simulation
Source: Nat Commun. 2021 Jan 25;12:573. doi: 10.1038/s41467-020-20884-9 (PMC7835367; doi:10.1038/s41467-020-20884-9)
Supplement: Supplementary file 1 — Supplementary Information [file 41467_2020_20884_MOESM1_ESM.pdf]

Supplementary Information

Nanoscale Characterization of the Biomolecular Corona by Cryo-Electron Microscopy, Cryo-Electron Tomography, and Image Simulation

Sara Sheibani[1*,#], Kaustuv Basu[2*], Ali Farnudi[3], Aliakbar Ashkarran[5], Muneyoshi Ichikawa[1], John F. Presley[1], Khanh Huy Bui[1,2], Mohammad Reza Ejtehadi[3,4,#], Hojatollah Vali[1,2], and Morteza Mahmoudi[5,#]

[1]Department of Anatomy & Cell Biology, McGill University, Montreal, Quebec H3A 0C7, Canada

[2]Facility for Electron Microscopy Research, McGill University, Montreal, Quebec H3A 0C7, Canada

[3]Department of Physics, Sharif University of Technology, Tehran, Iran

[4]School of Nano Science, Institute for Research in Fundamental Sciences (IPM), Tehran, Iran

[5]Department of Radiology and Precision Health Program, College of Human Medicine, Michigan State University, East Lansing, USA

* Co-authors

[#] Corresponding authors: (MRE) email: ejtehadi@sharif.edu; (SS) email: sara.sheibani@mcgill.ca; (MM) email: mahmou22@msu.edu

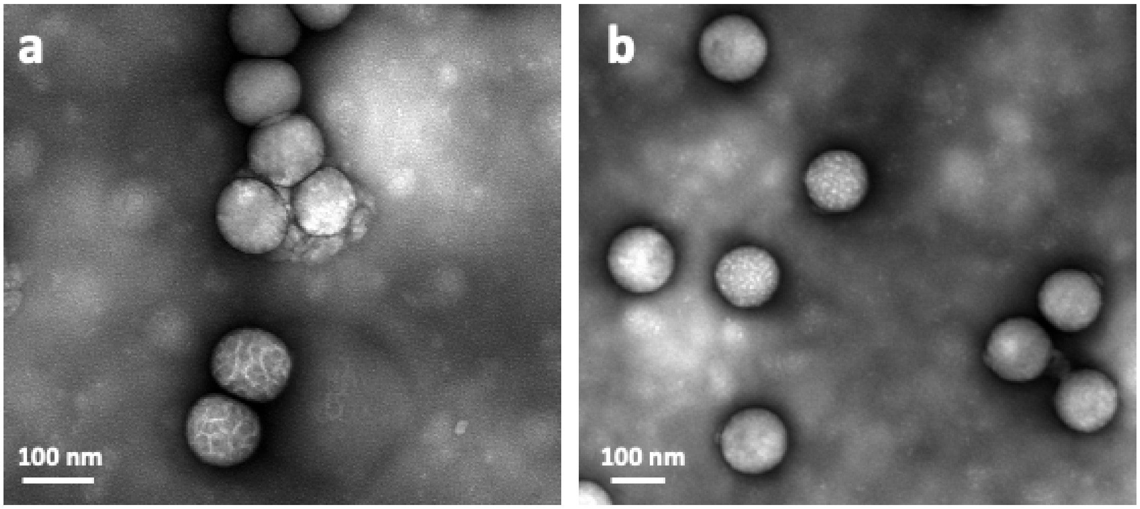

Supplementary Figure 1. (a) Protein corona (hard and soft) formation on the surface on nanoparticles (no wash) (b) hard protein corona formation on the surface of nanoparticle (with washing step).

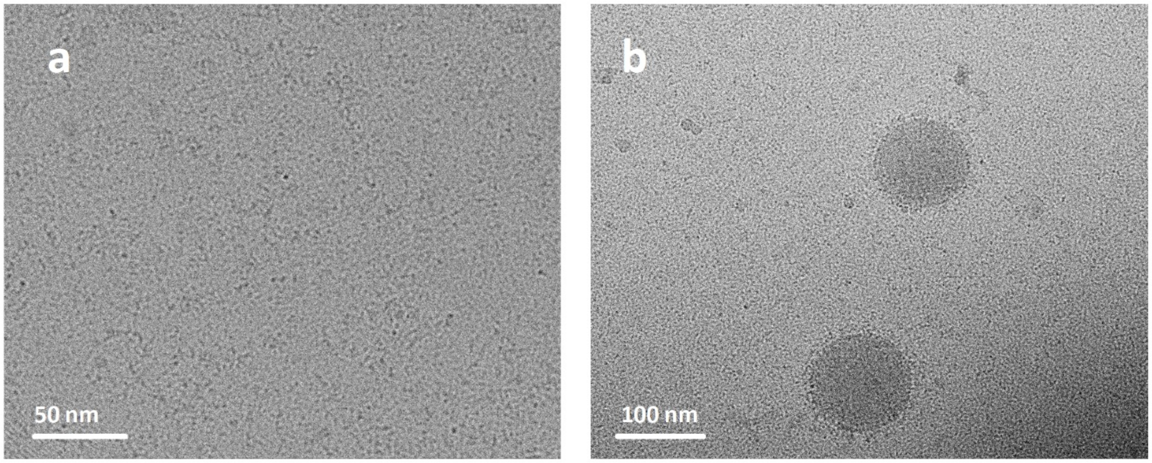

Supplementary Figure 2. Cryo-EM images of the PS-COOH NPs (a) original plasma diluted in Sorensen's phosphate buffer to 50%. There is little evidence for presence of large cluster and aggregate; (b) NPs with corona in 2% solution showing the distribution of biomolecules and their association with the surface of the NPs. Similar to the plasma, there is little evidence for large clusters.

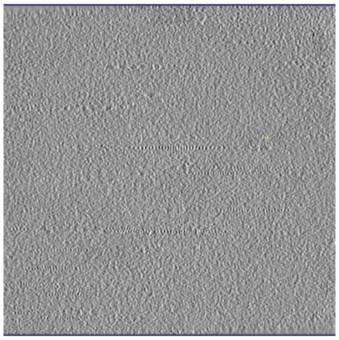
1

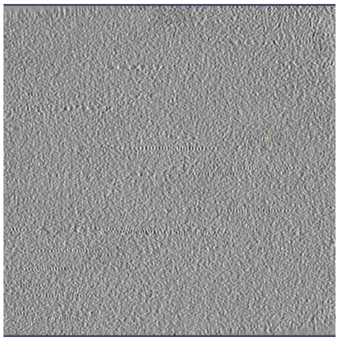
2

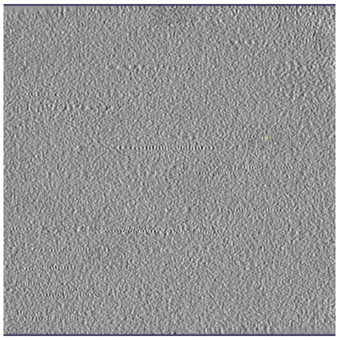
3

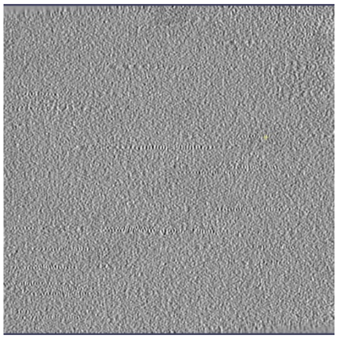
4

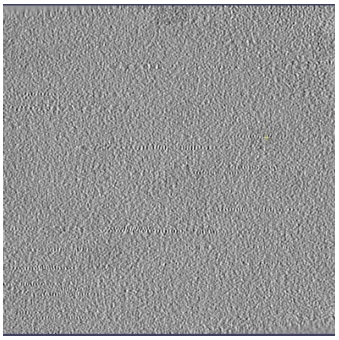
5

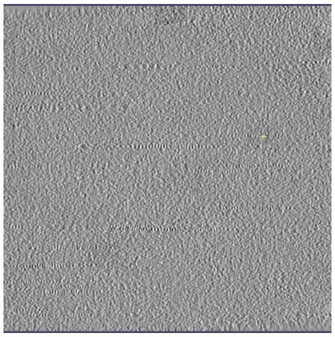
6

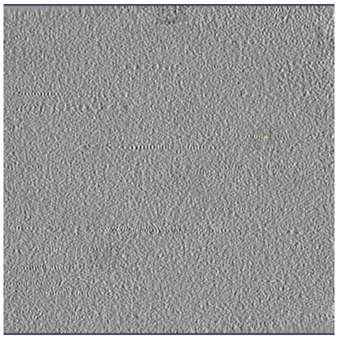
7

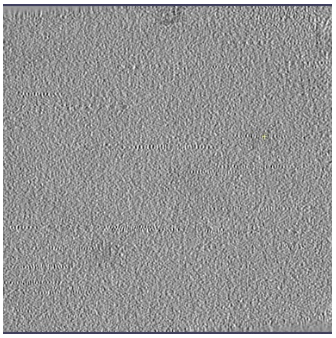
8

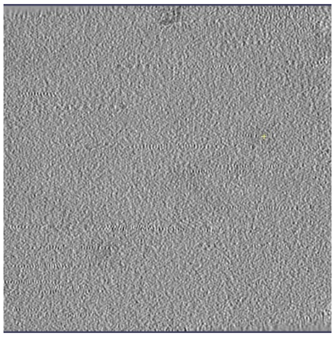
9

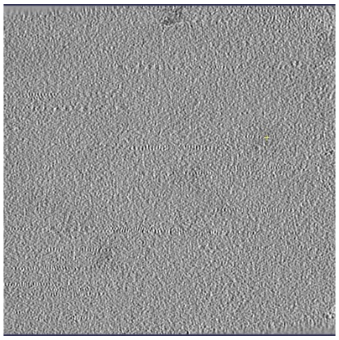
10

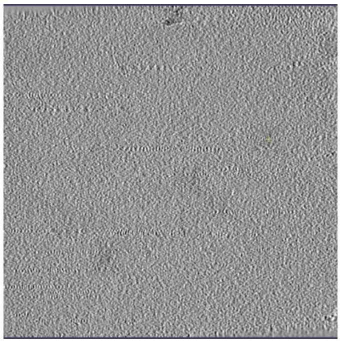
11

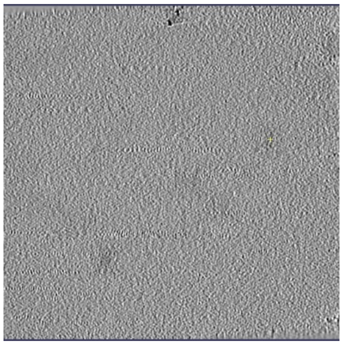
12

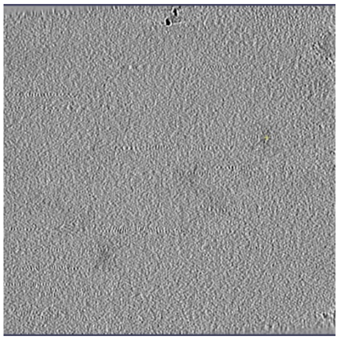
13

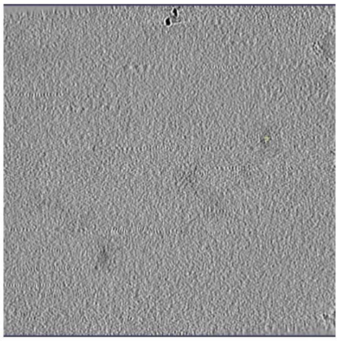
14

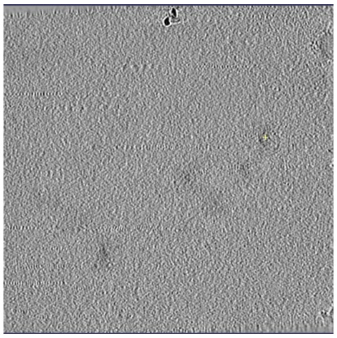
15

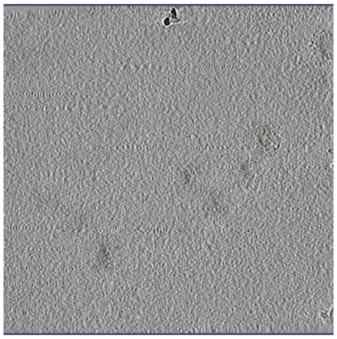
16

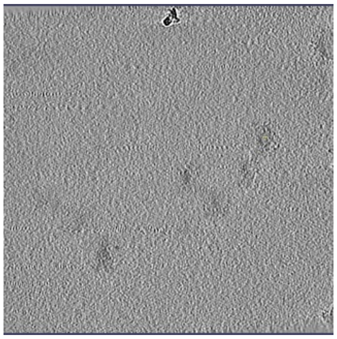
17

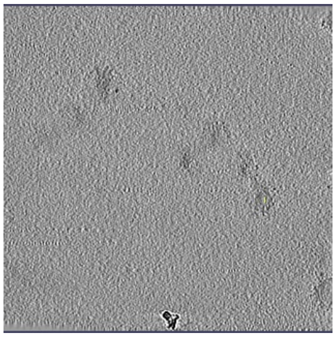
18

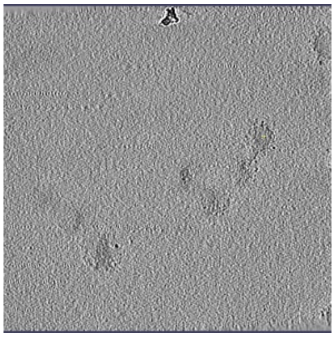
19

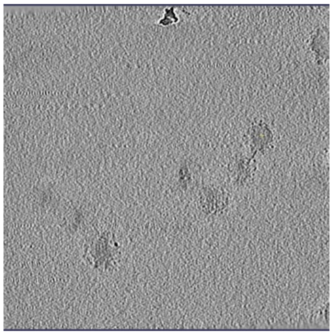
20

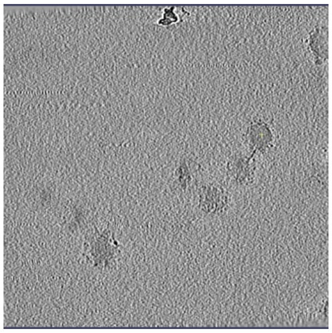
21

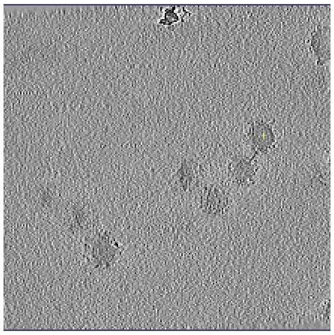
22

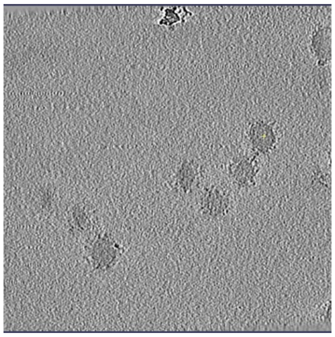
23

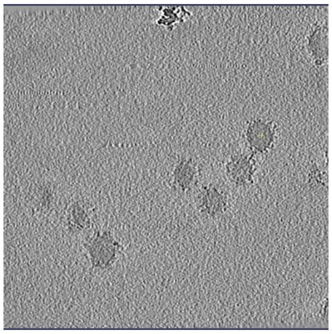
24

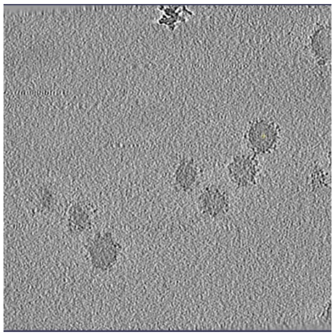
25

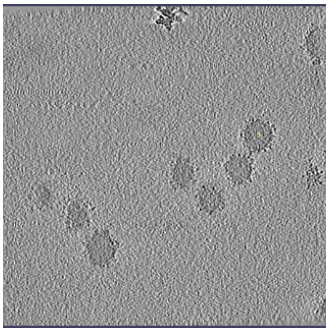
26

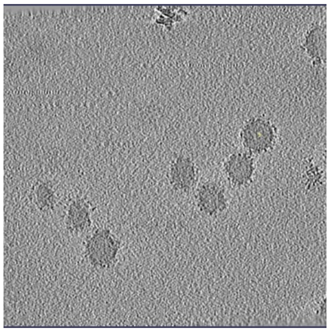
27

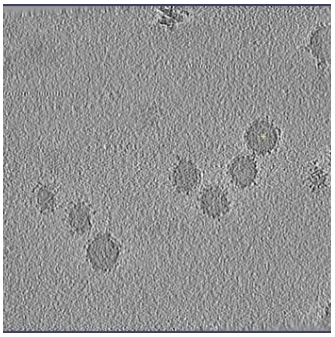
28

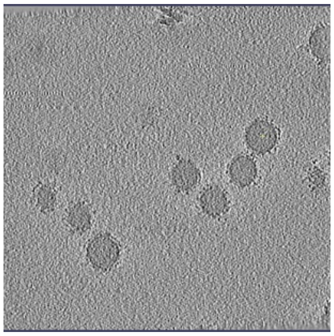
29

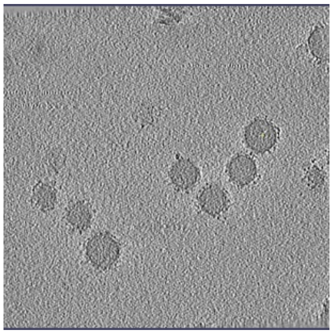
30

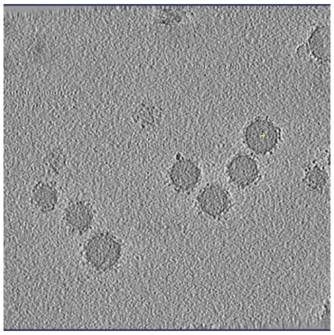
31

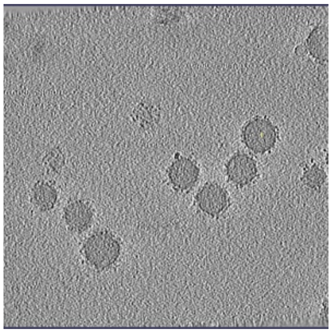
32

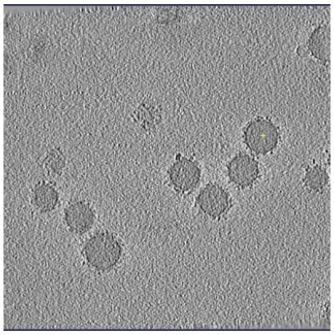
33

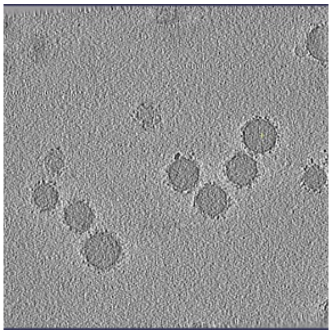
34

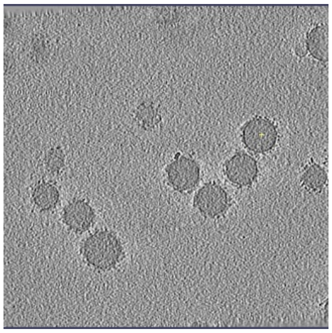
35

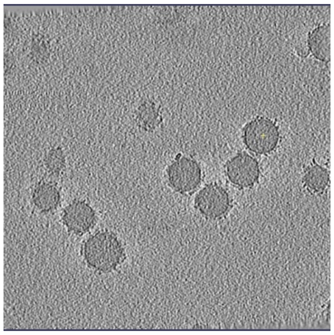
36

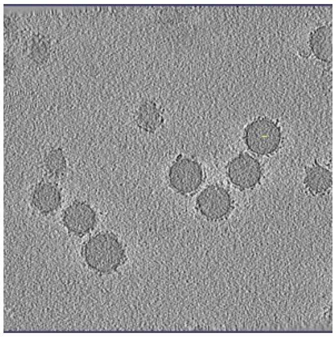

37

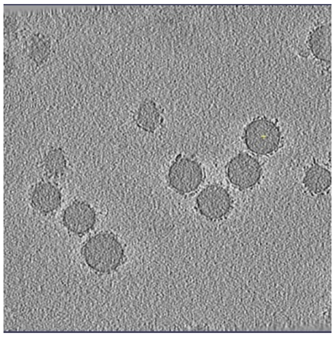

38

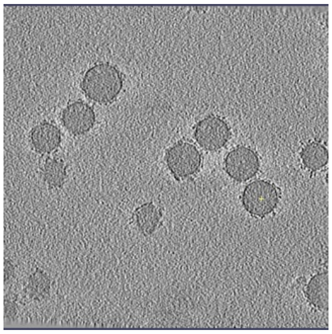

39

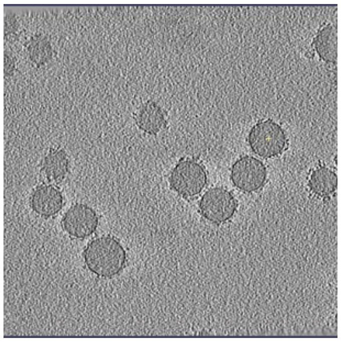

40

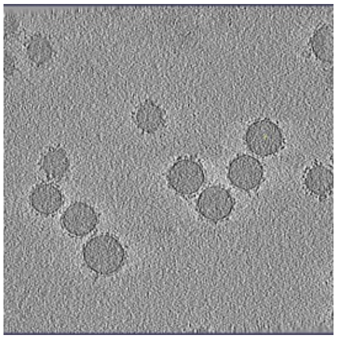

41

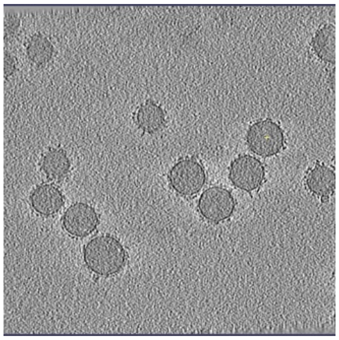

42

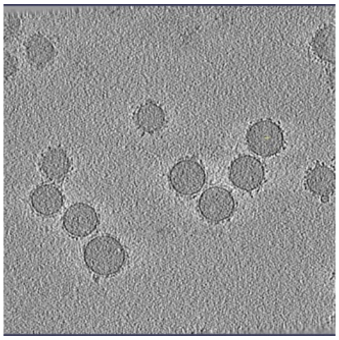

43

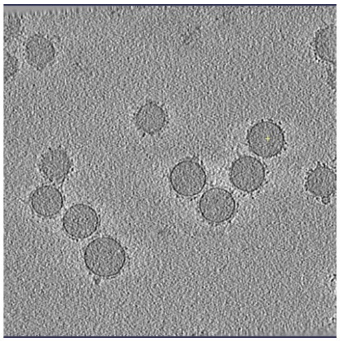

44

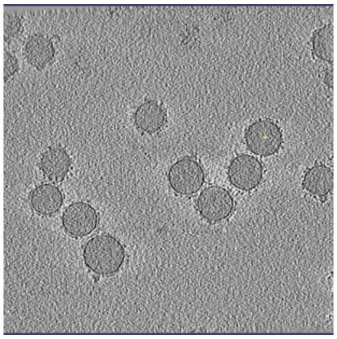

45

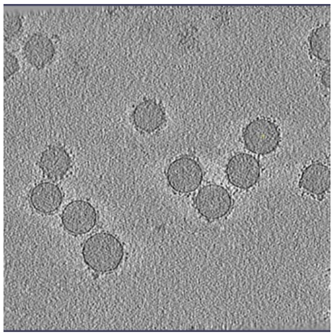

46

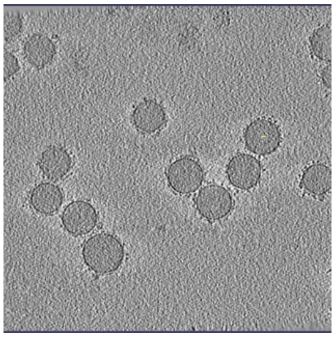

47

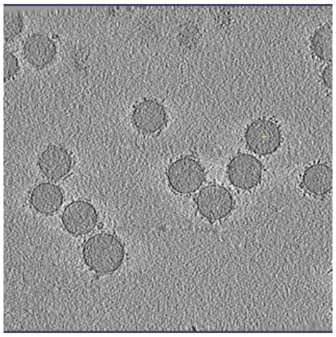

48

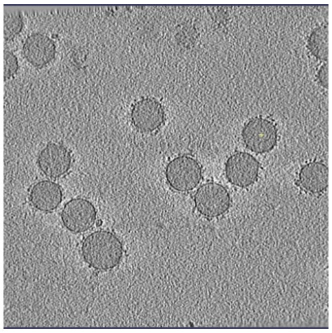

49

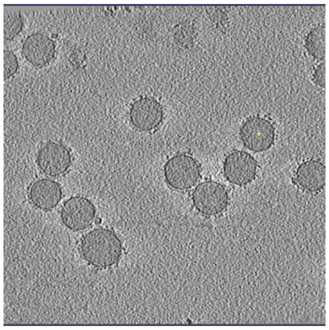

50

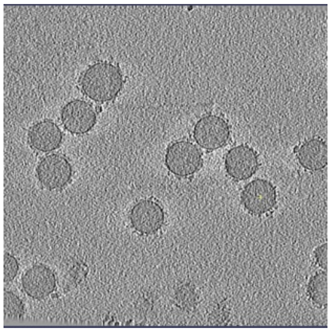

51

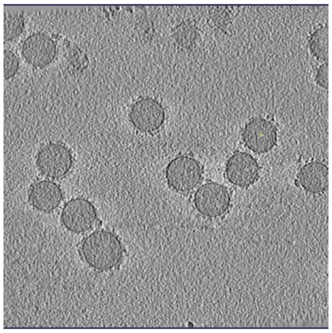

52

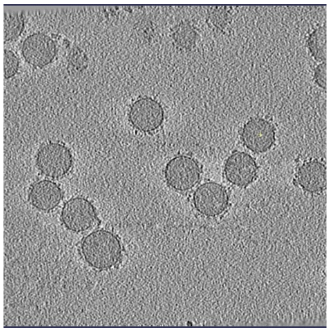

53

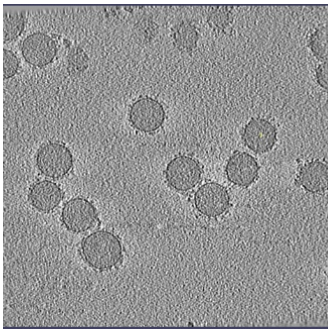

54

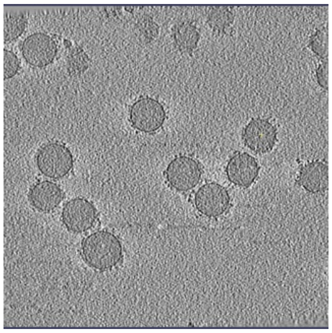

55

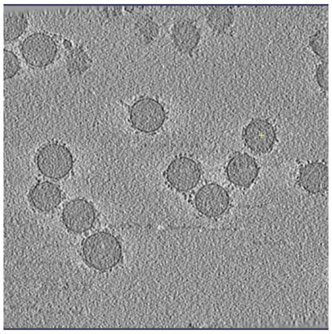

56

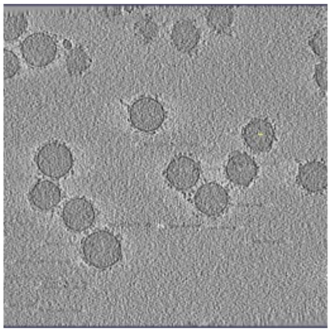

57

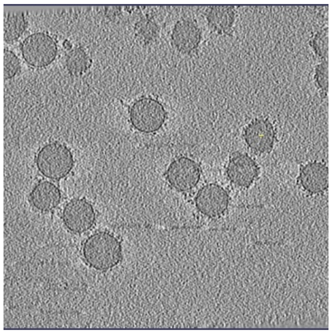

58

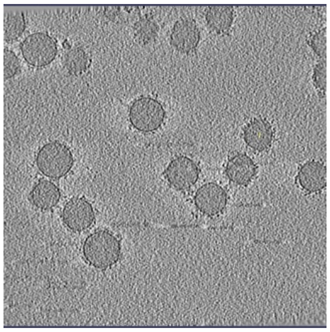

59

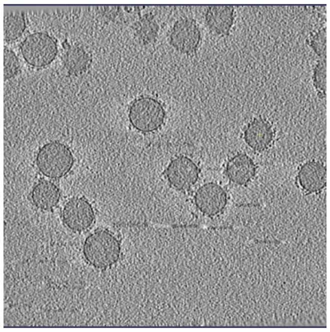

60

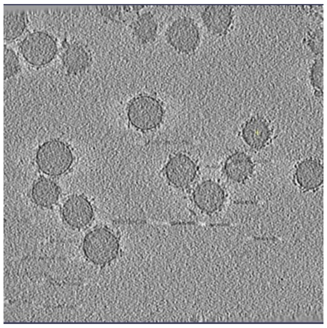

61

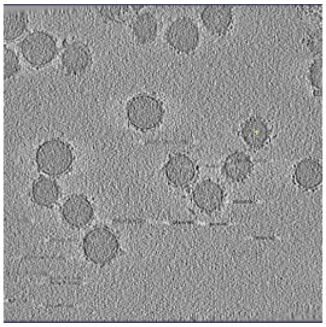

62

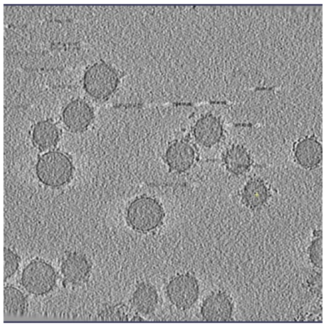

63

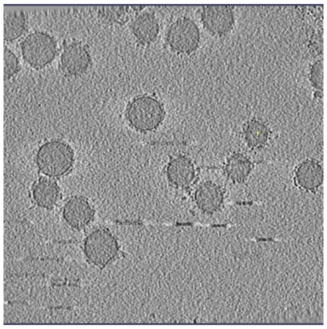

64

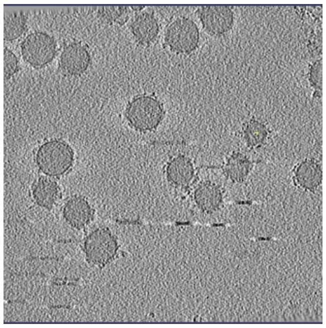

65

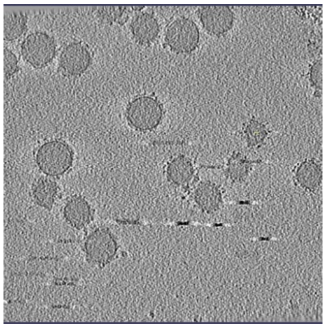

66

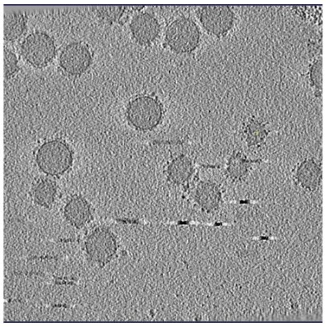

67

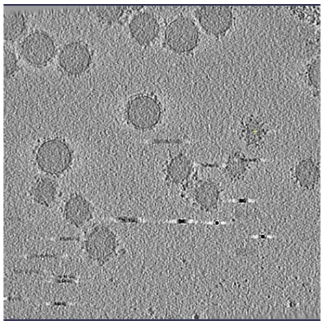

68

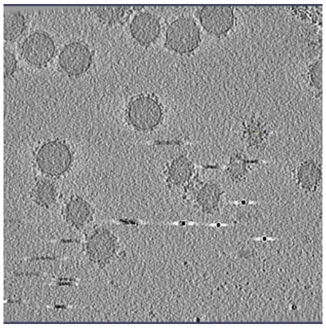

69

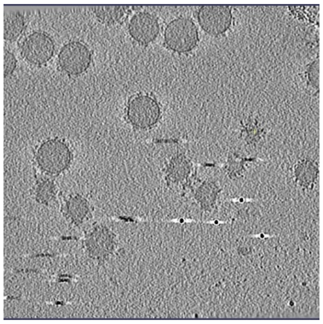

70

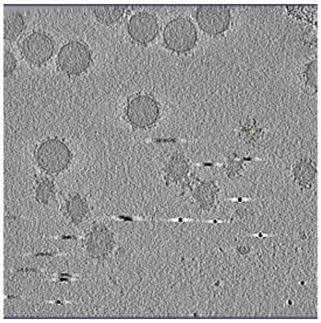

71

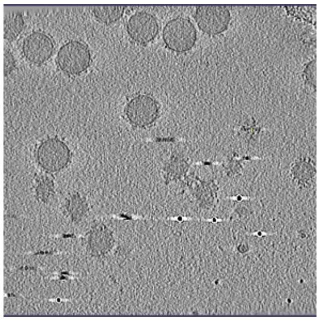

72

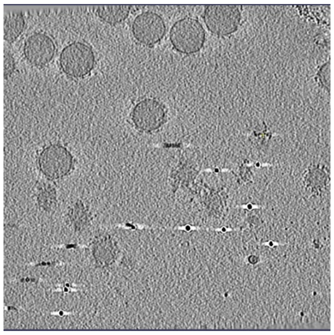

73

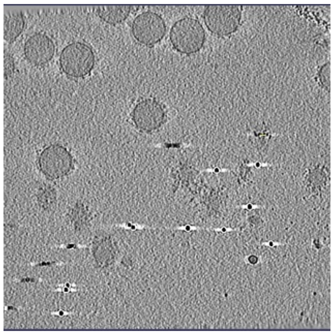

74

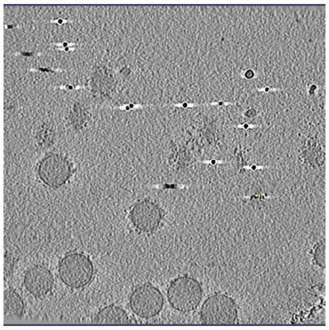

75

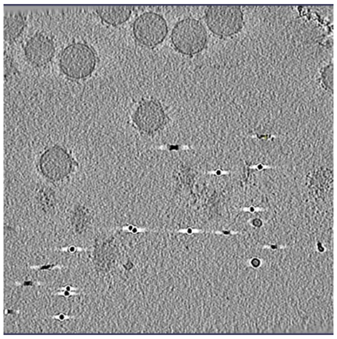

76

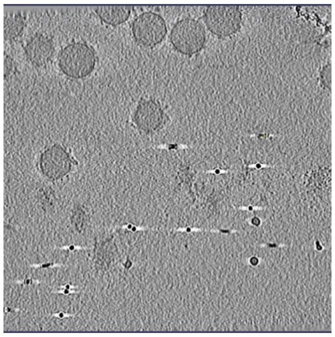

77

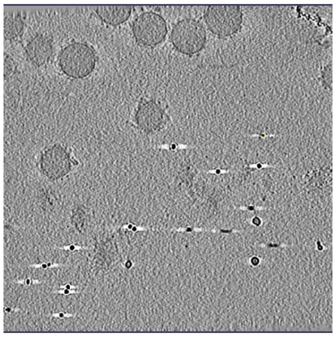

78

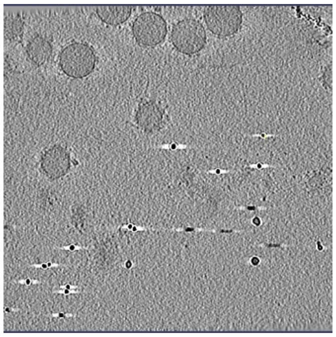

79

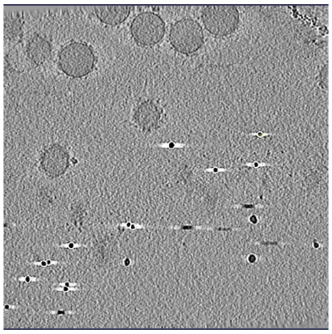

80

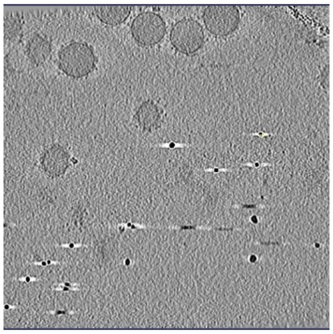

81

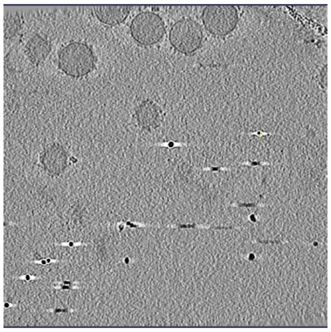

82

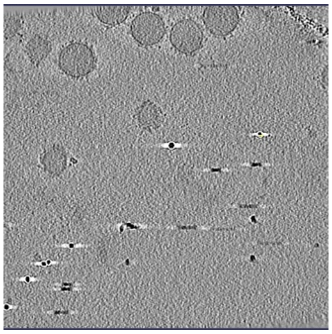

83

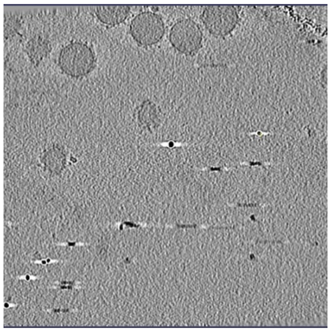

84

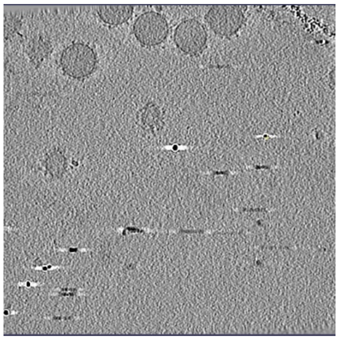
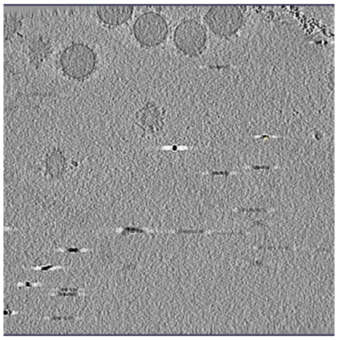
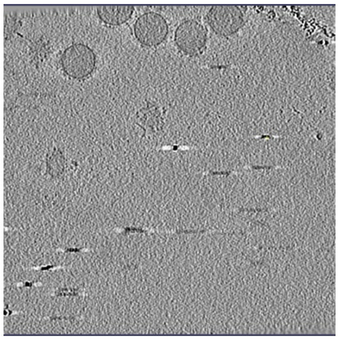

85 86 87

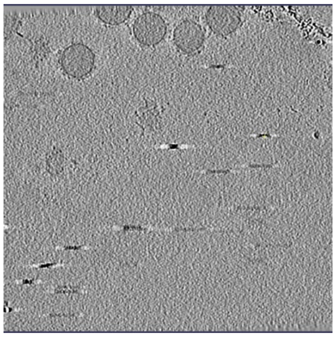
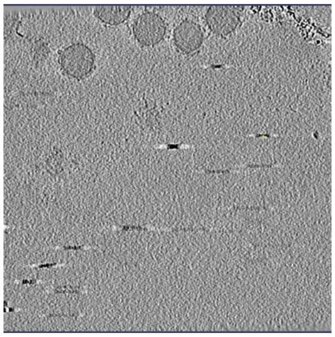
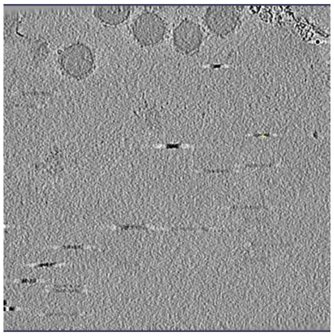

88 89 90

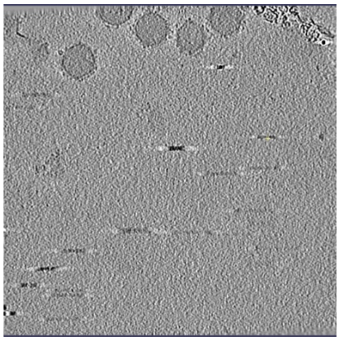
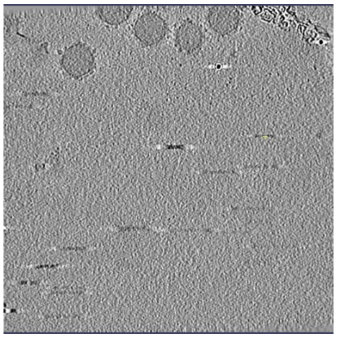
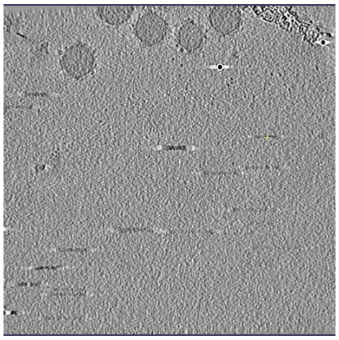

91 92 93

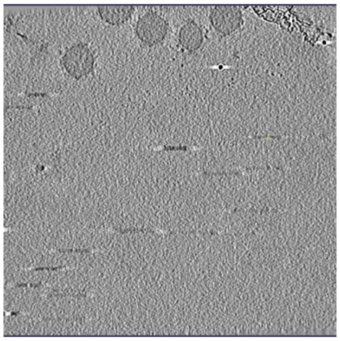
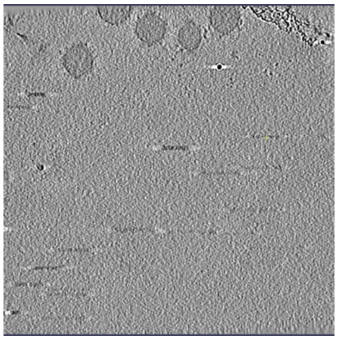
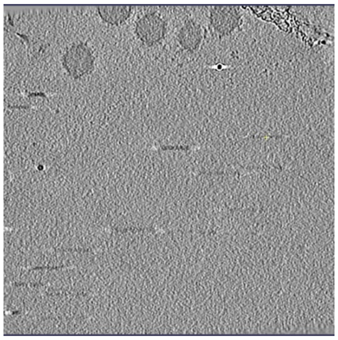

94 95 96

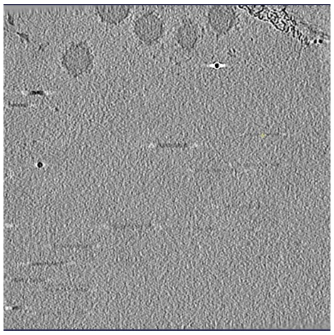
97

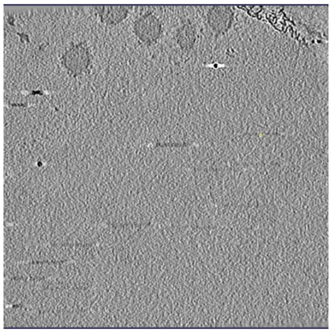
98

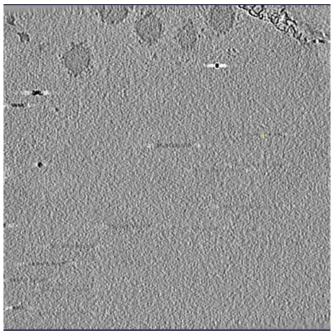
99

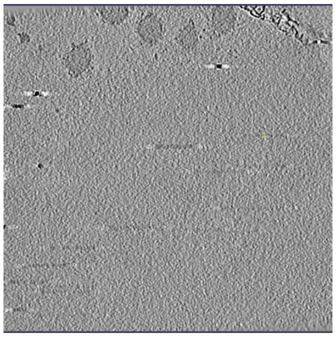
100

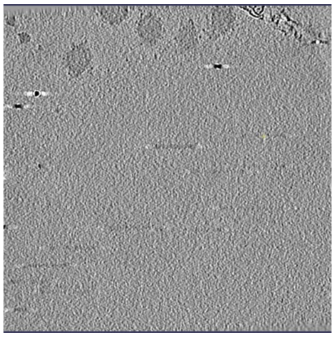
101

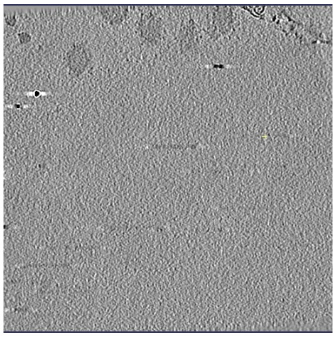
102

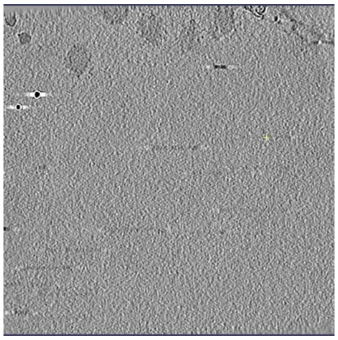
103

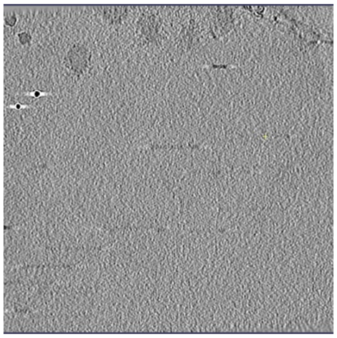
104

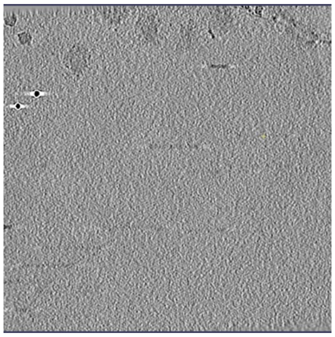
105

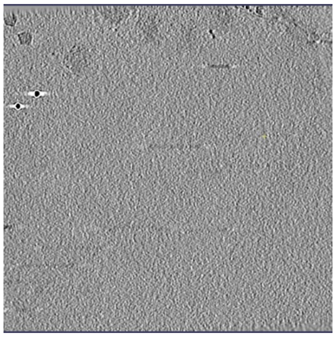
106

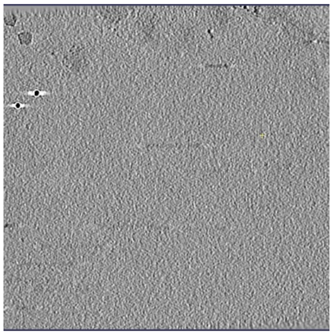
107

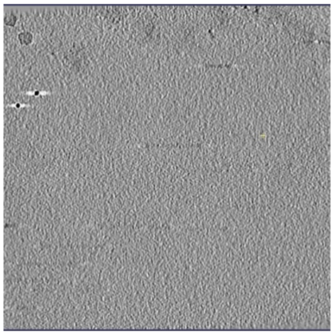
108

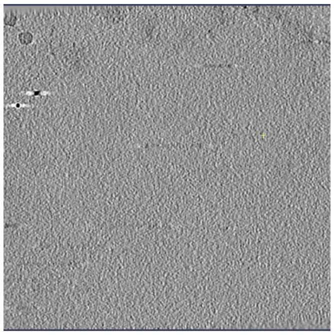

109

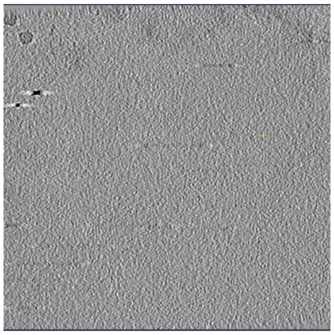

110

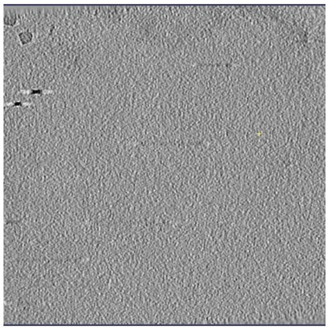

111

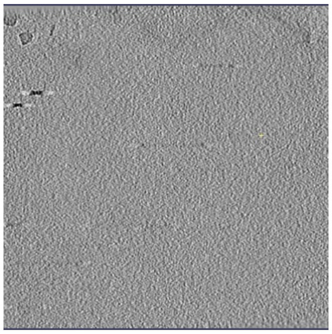

112

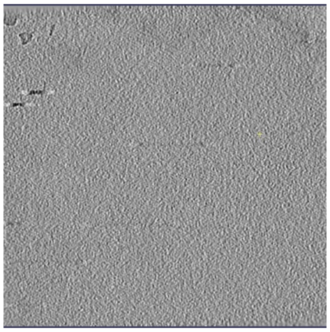

113

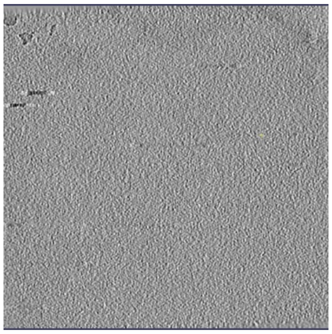

114

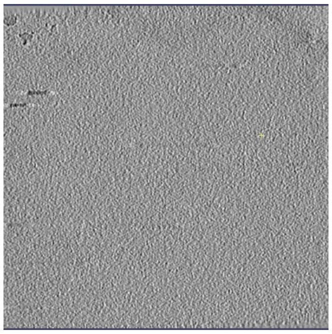

115

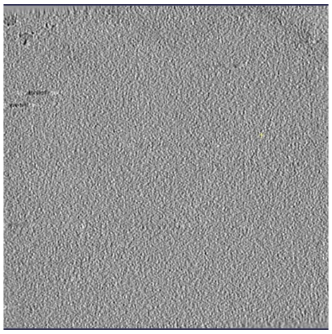

116

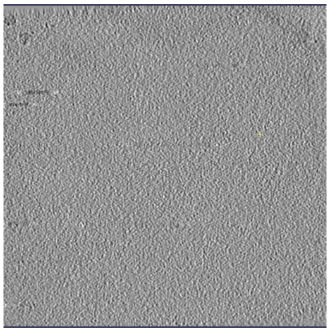

117

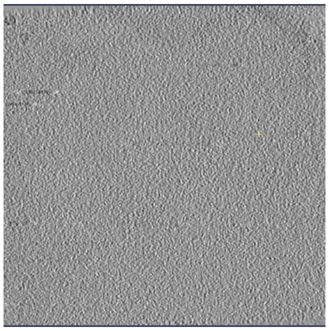

118

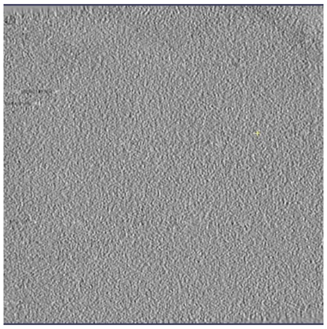

119

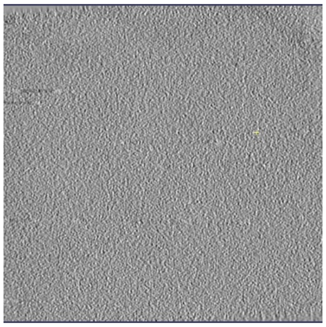

120

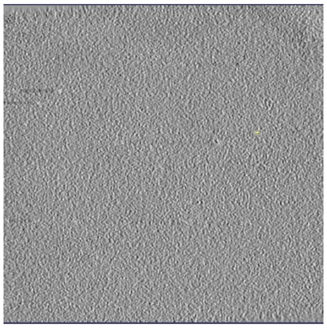

121

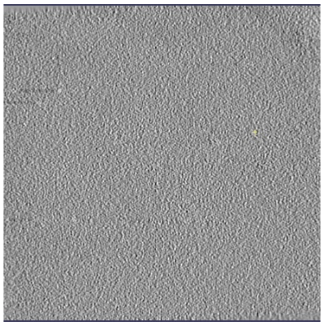

122

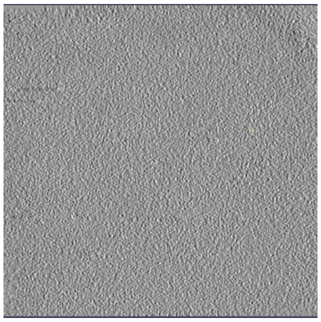

123

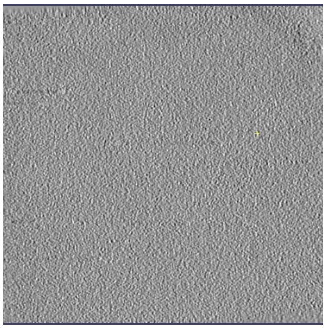

124

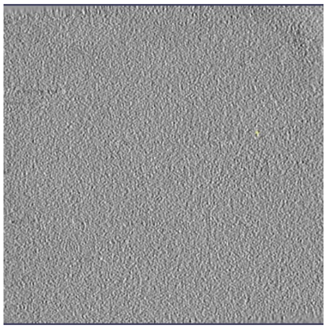

125

Supplementary Figure 3. TEM images showing the 3D tomographic volume slices of 10% corona coated NPs. The size of each image panel is 1μm

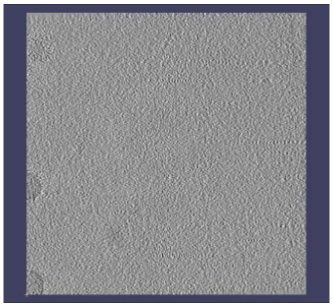

1

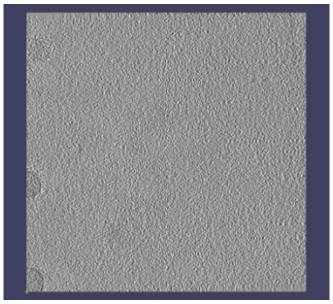

2

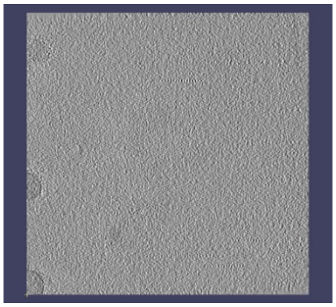

3

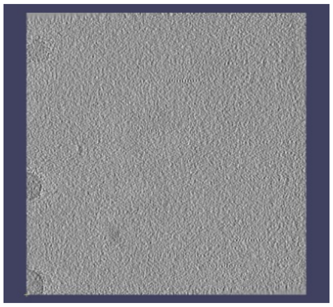

4

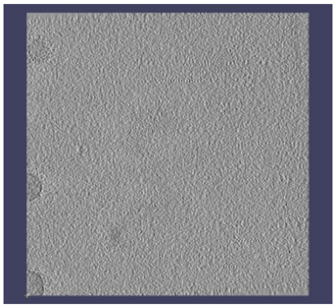

5

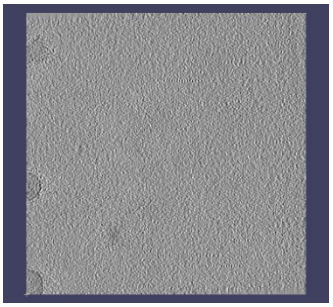

6

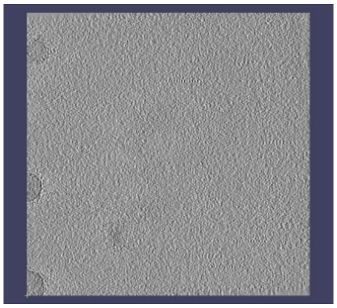

7

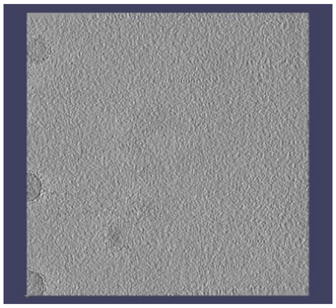

8

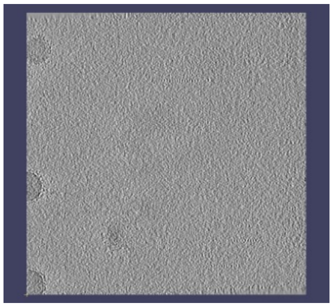

9

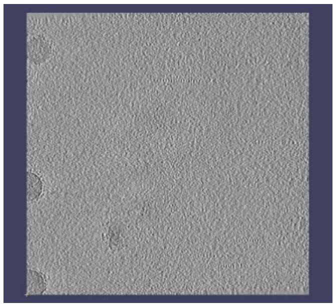

10

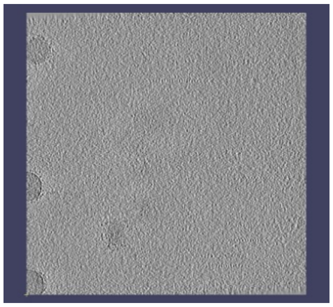

11

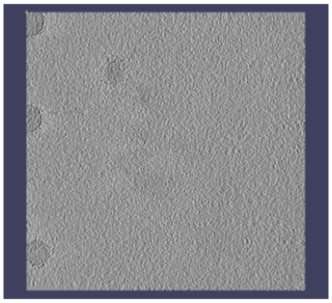

12

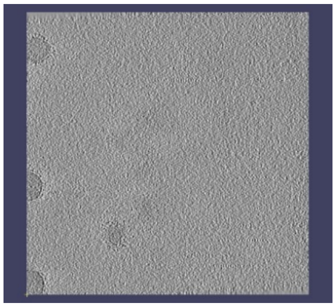

13

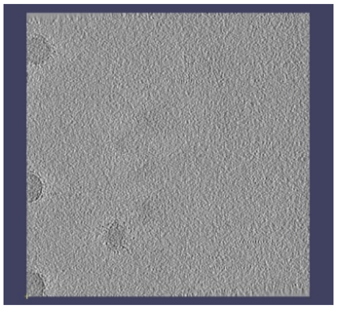

14

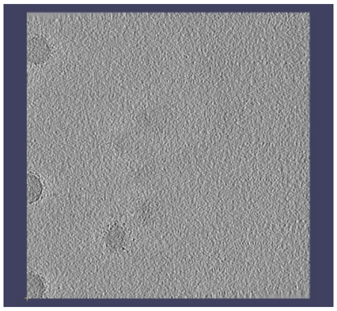

15

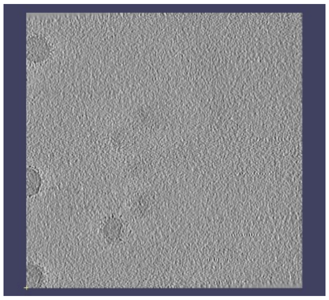

16

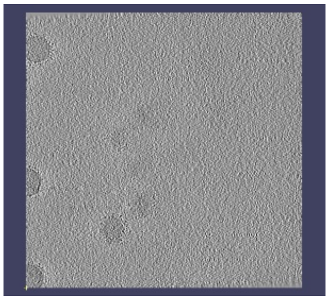

17

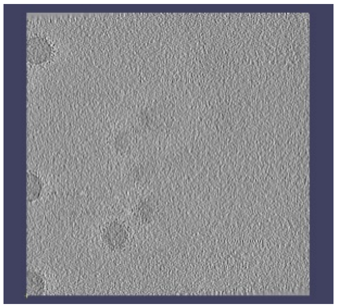

18

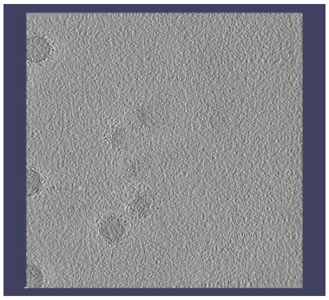

19

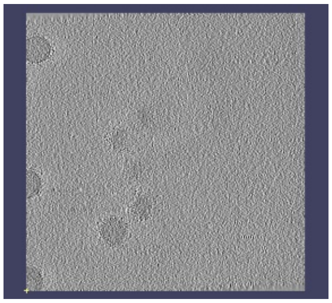

20

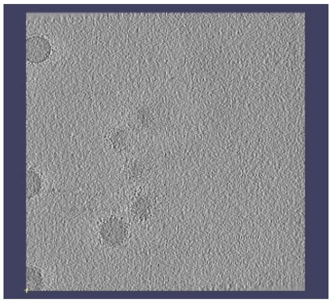

21

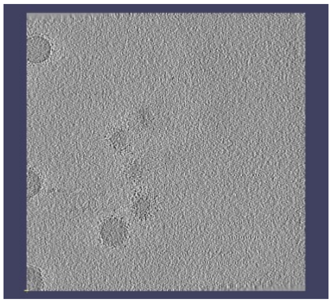

22

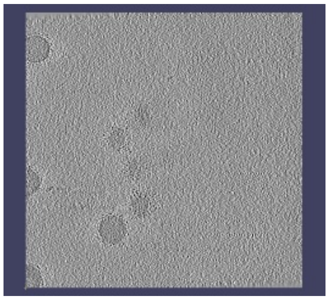

23

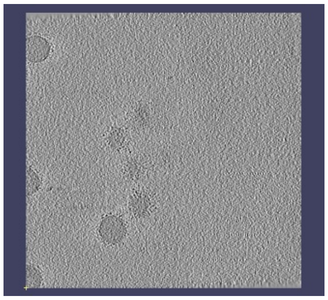

24

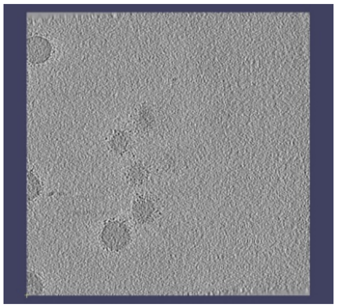

25

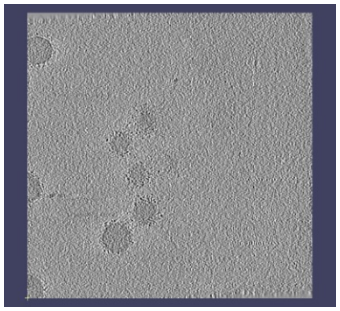

26

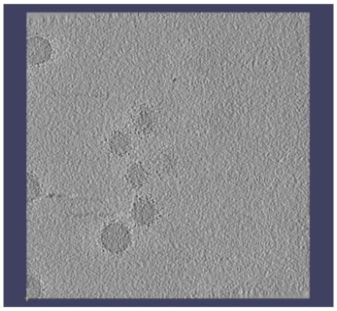

27

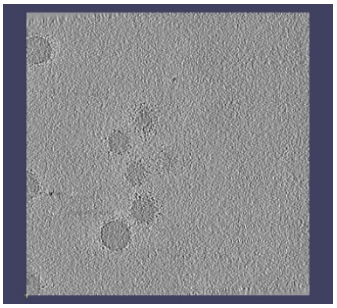

28

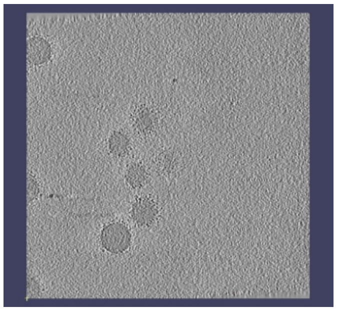

29

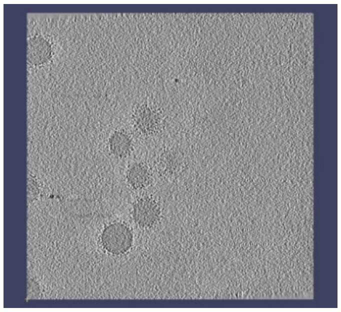

30

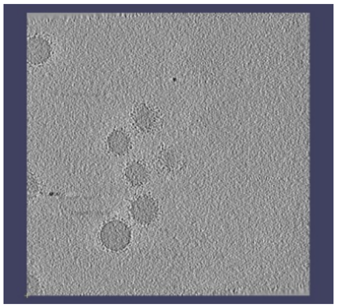

31

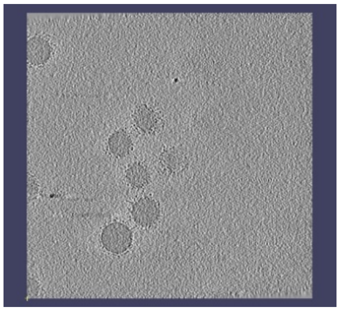

32

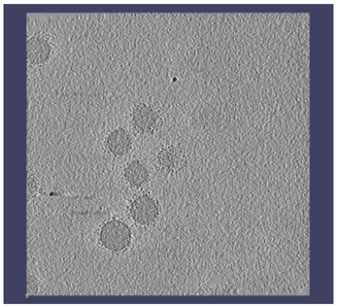

33

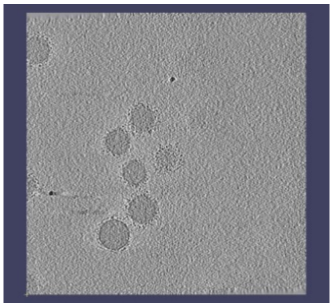

34

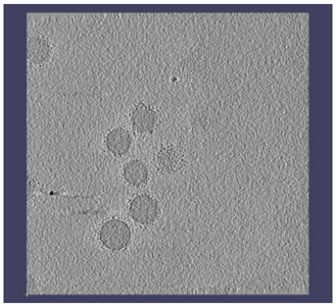

35

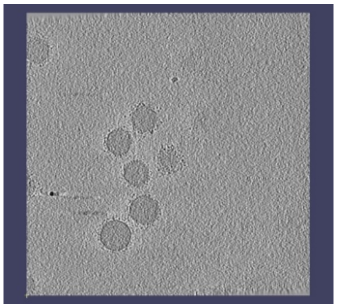

36

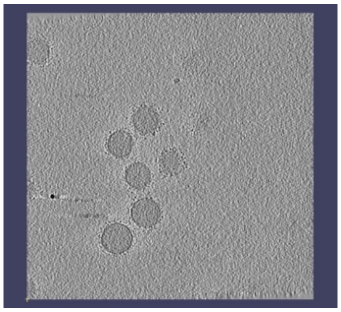

37

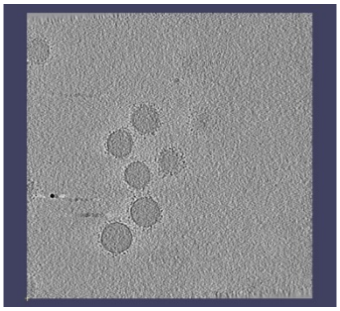

38

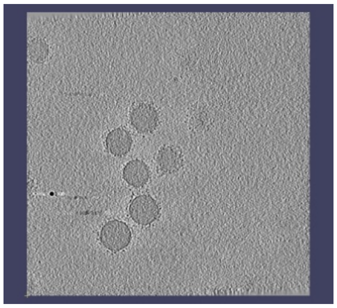

39

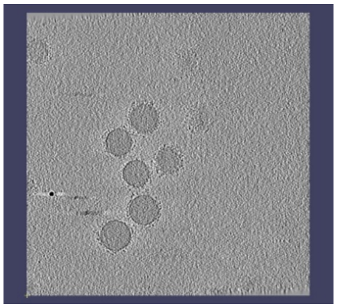

40

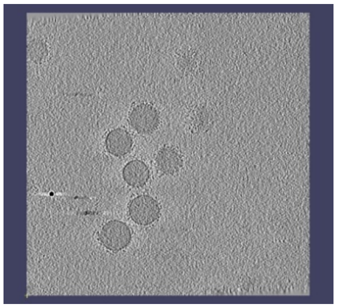

41

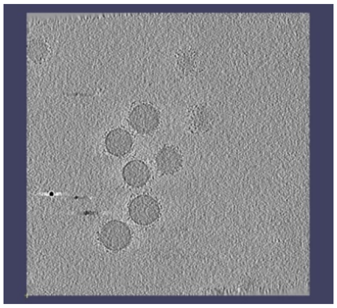

42

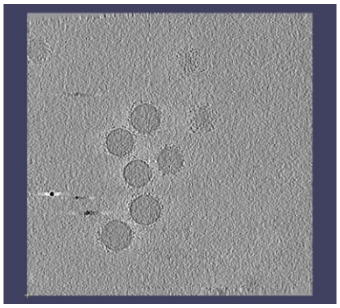

43

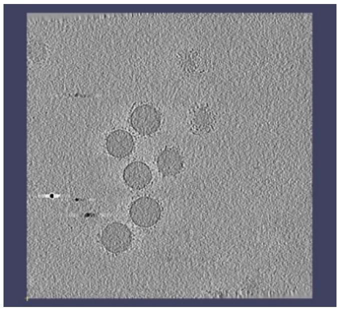

44

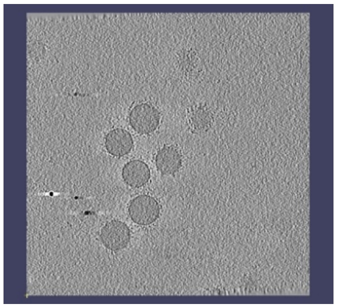

45

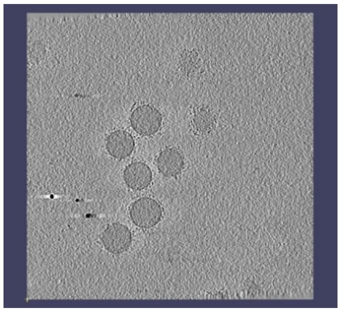

46

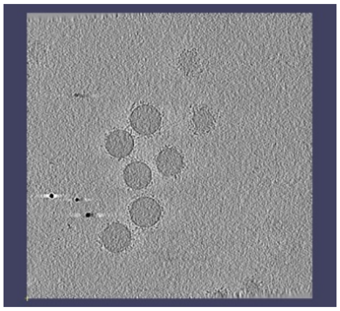

47

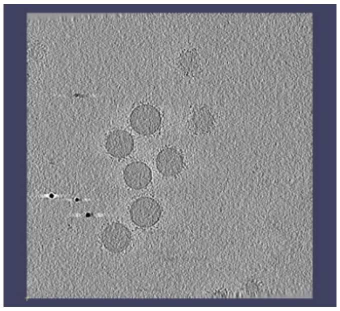

48

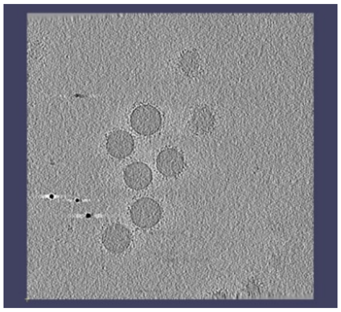

49

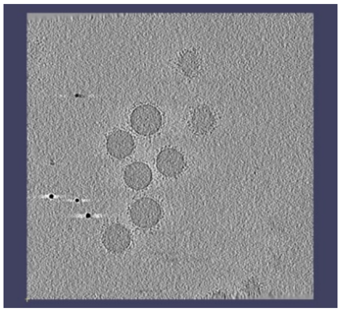

50

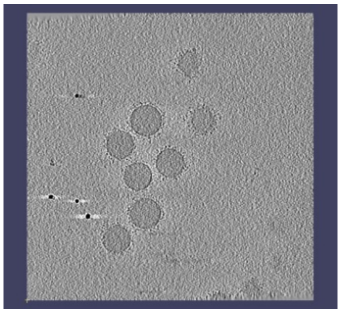

51

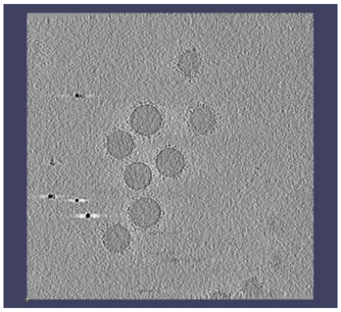

52

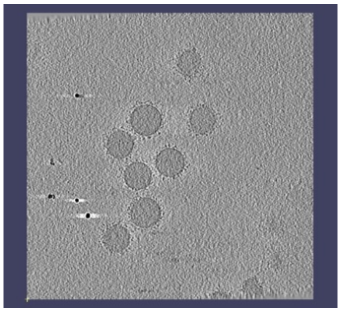

53

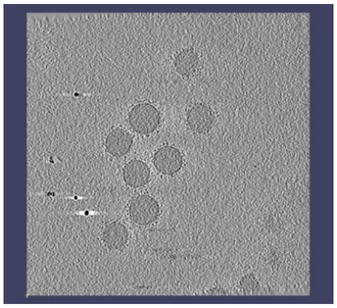

54

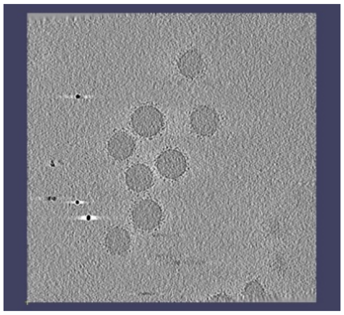

55

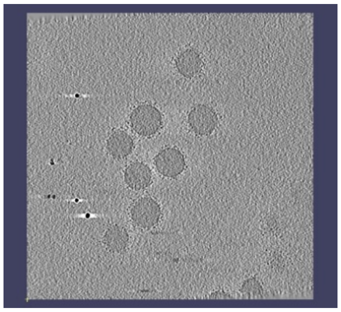

56

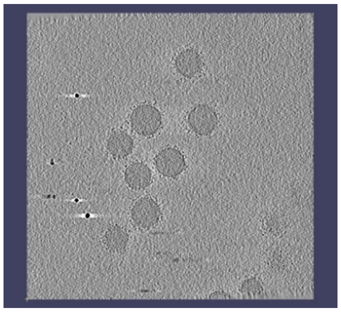

57

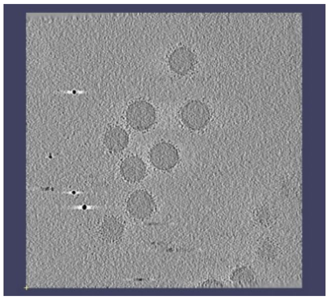

58

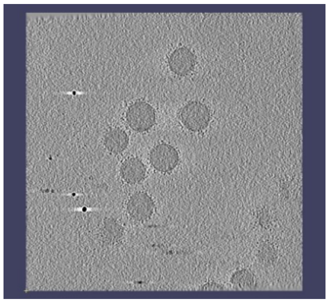

59

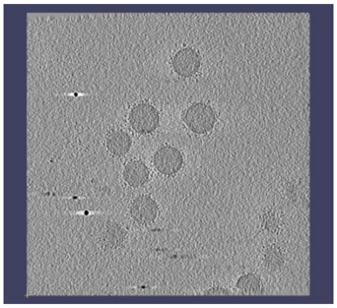

60

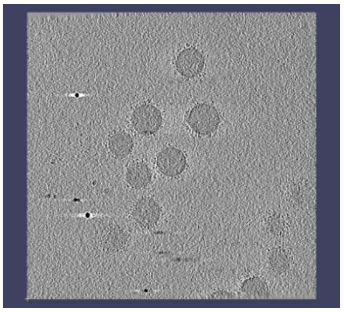
61

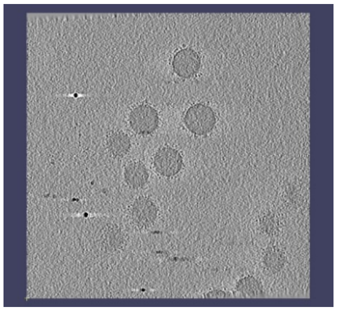
62

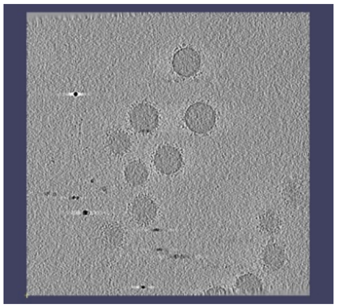
63

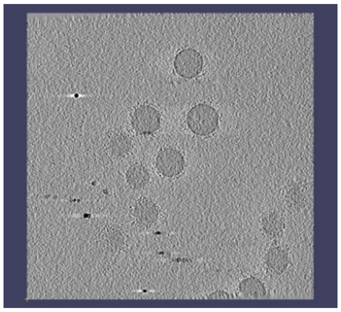
64

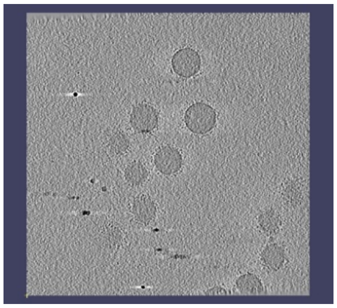
65

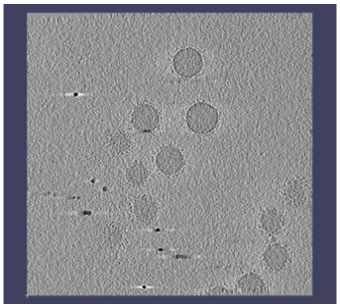
66

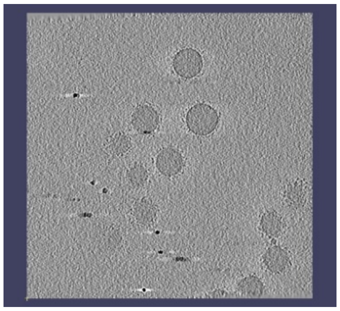
67

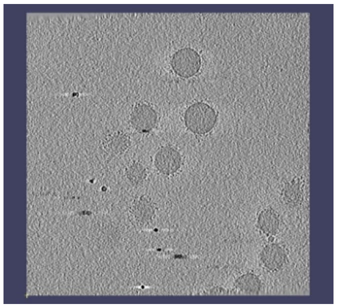
68

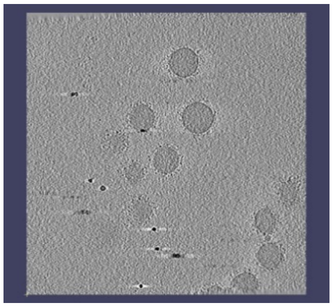
69

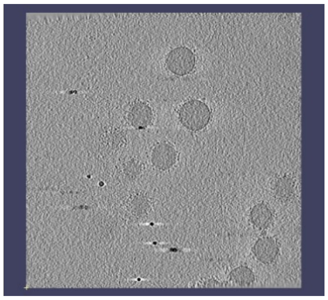
70

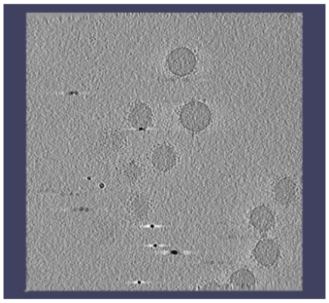
71

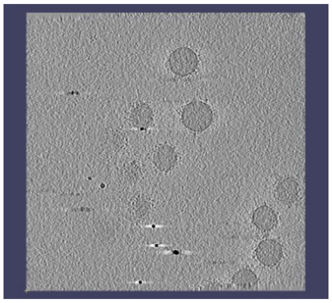
72

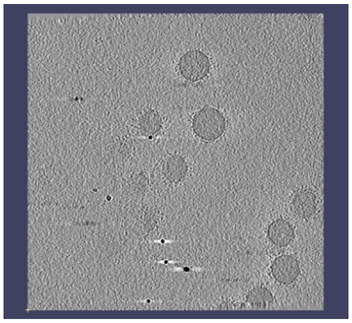

73

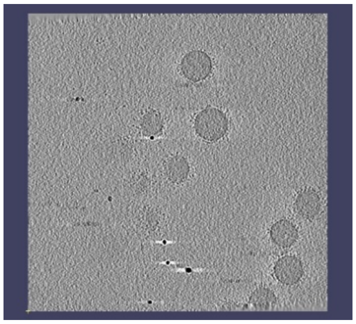

74

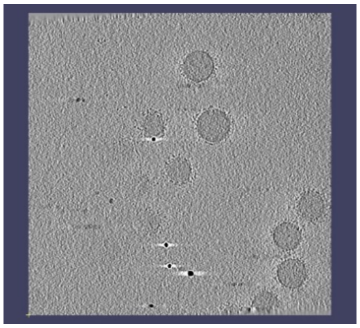

75

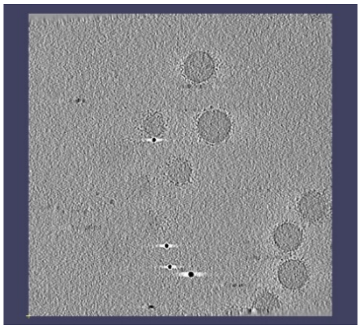

76

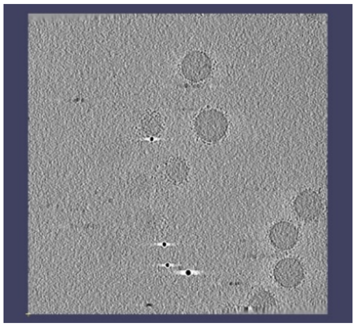

77

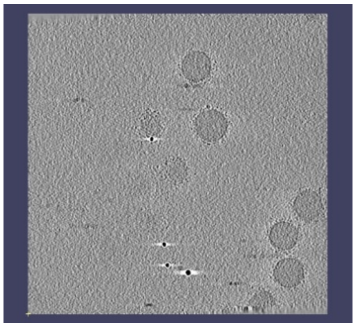

78

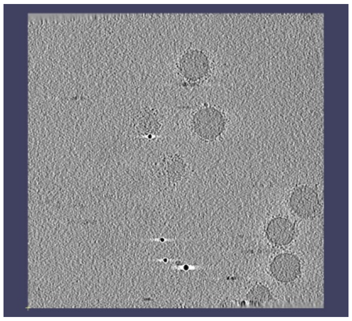

79

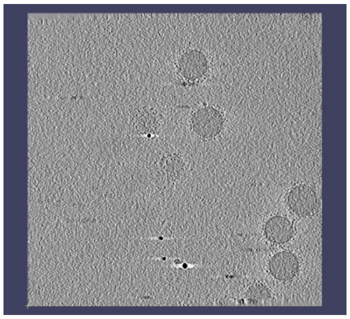

80

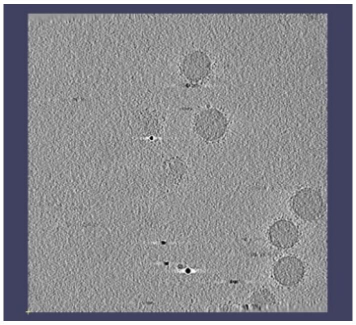

81

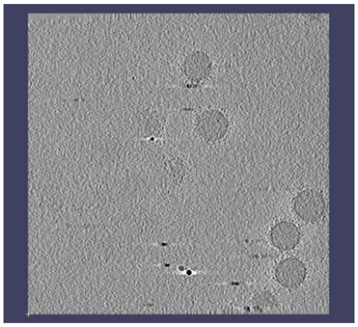

82

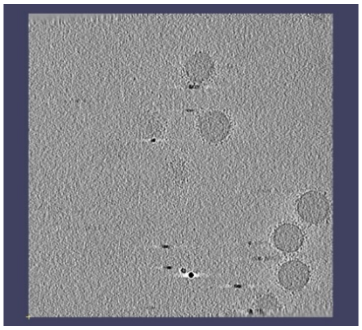

83

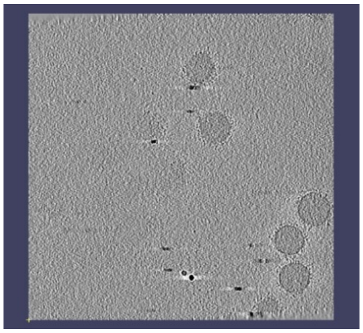

84

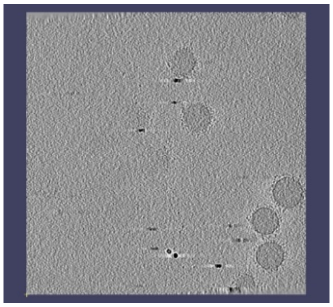

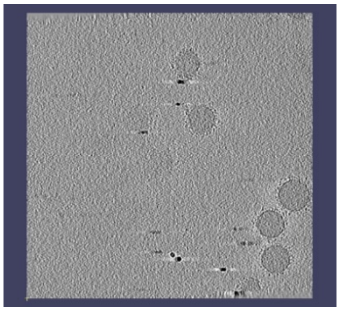

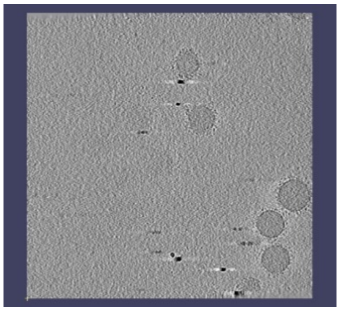

85

86

87

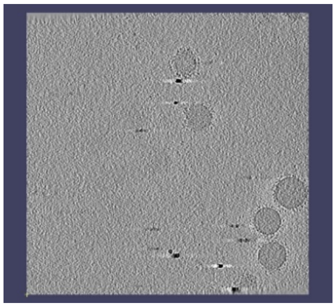

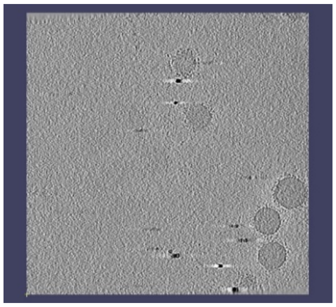

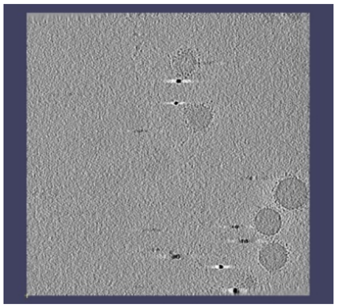

88

89

90

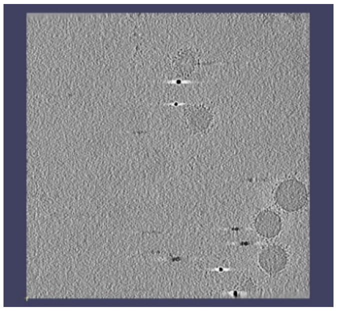

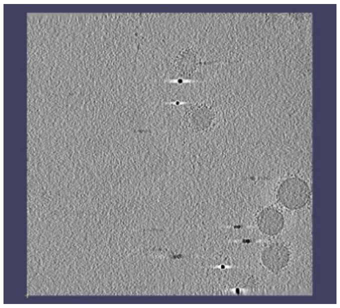

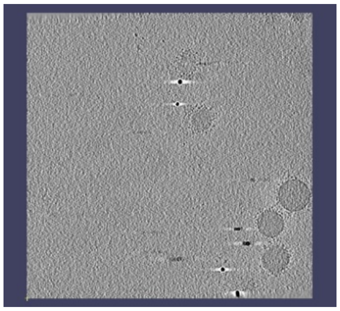

91

92

93

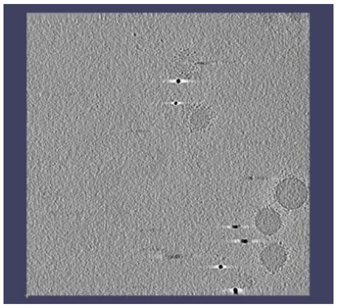

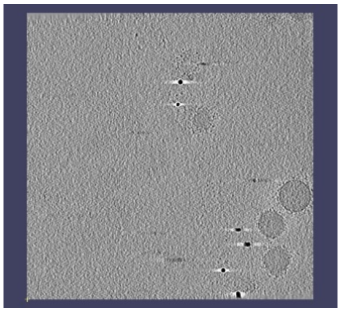

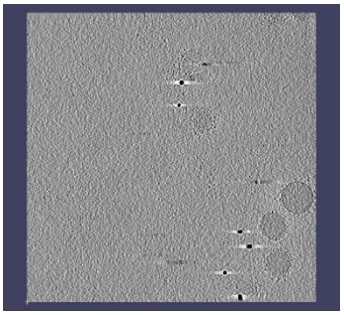

94

95

96

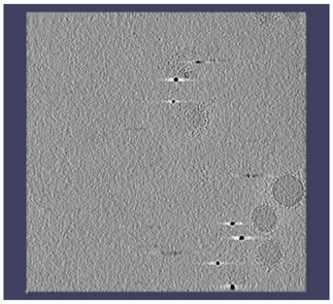

97

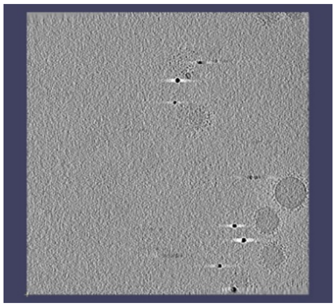

98

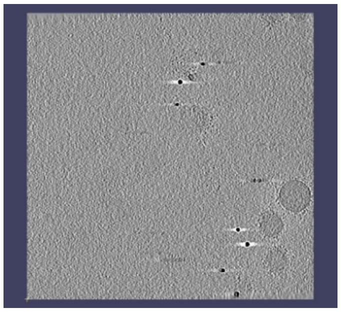

99

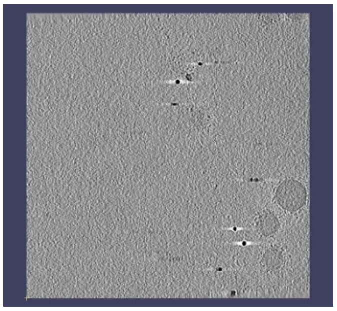

100

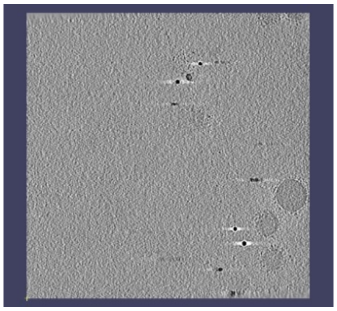

101

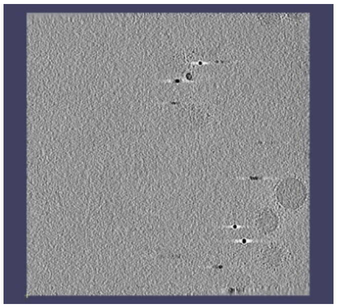

102

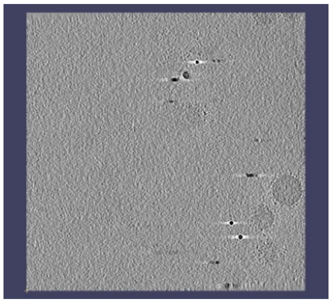

103

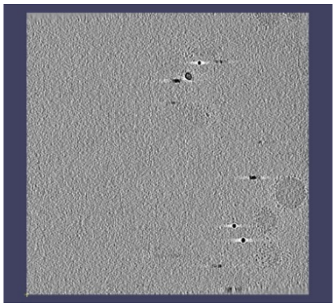

104

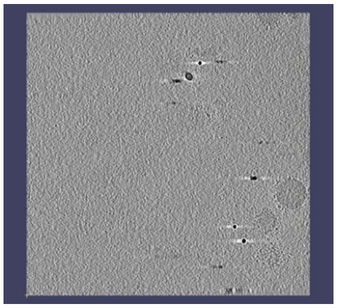

105

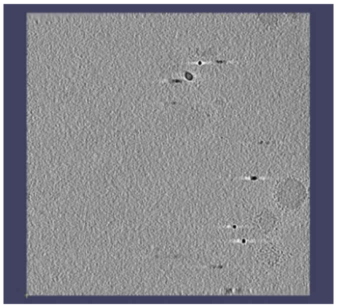

106

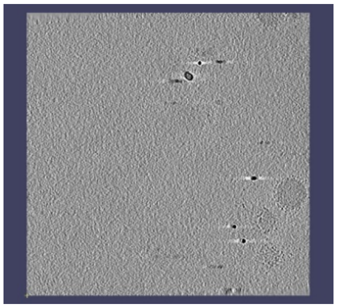

107

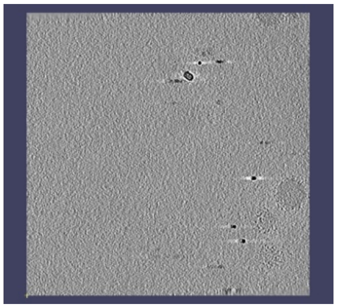

108

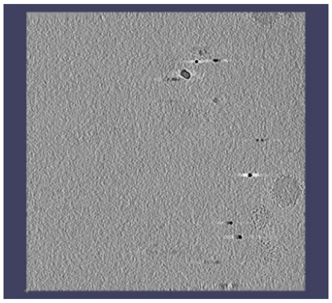

109

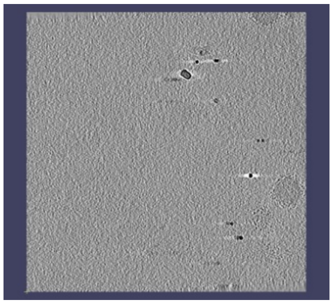

110

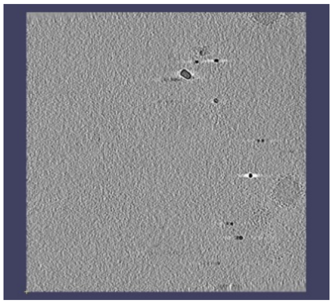

111

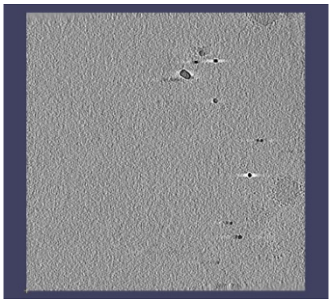

112

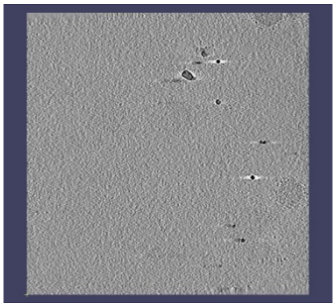

113

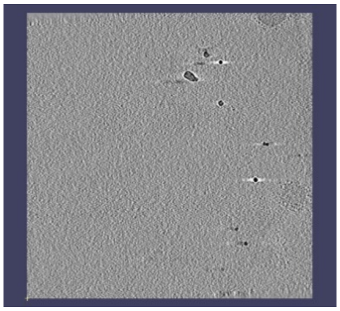

114

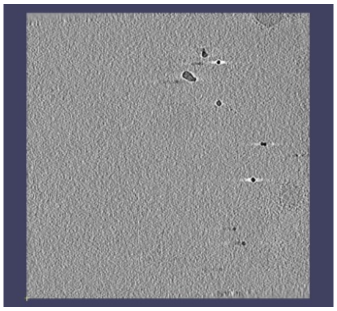

115

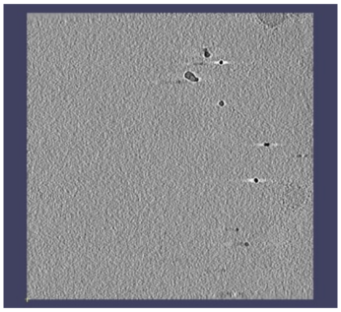

116

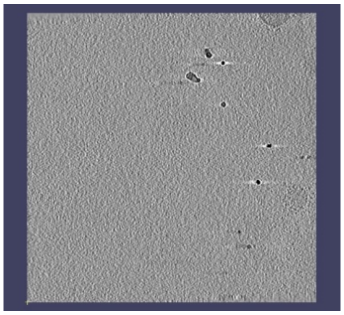

117

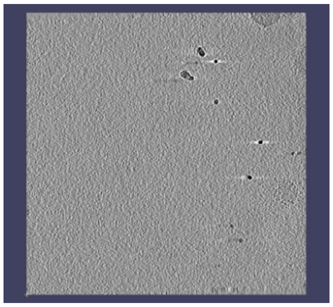

118

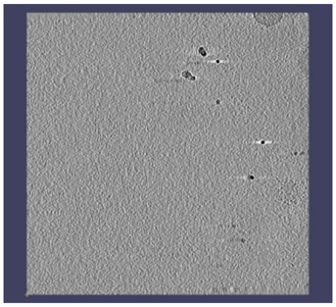

119

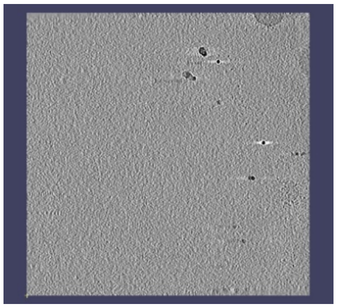

120

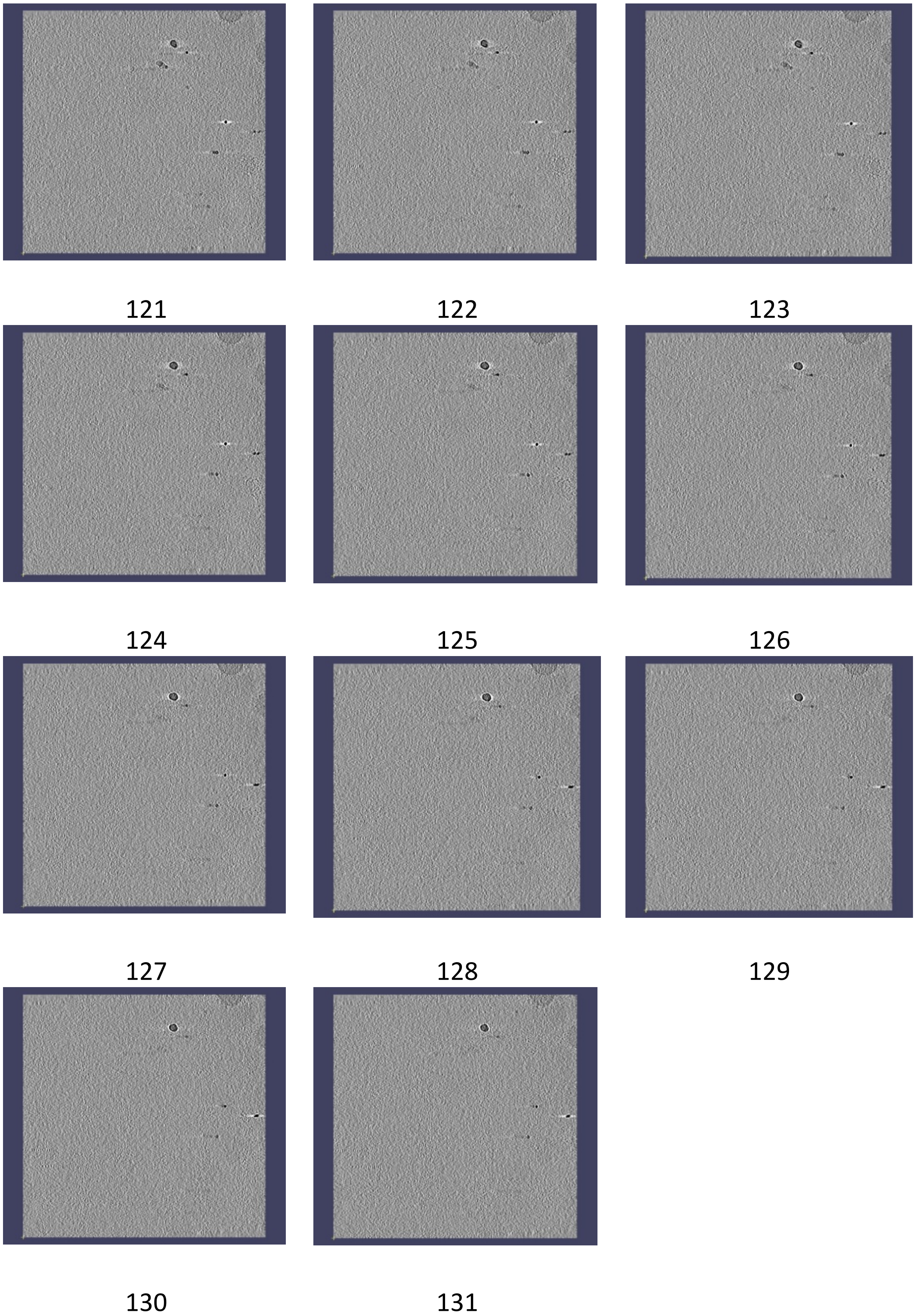

Supplementary Figure 4. TEM images showing the 3D tomographic volume slices of 50% corona coated NPs. The size of each image panel is 1μm.
