# Supplementary material for: Nanoscale characterization of the biomolecular corona by cryo-electron microscopy, cryo-electron tomography, and image simulation
Source: Nat Commun. 2021 Jan 25;12:573. doi: 10.1038/s41467-020-20884-9 (PMC7835367; doi:10.1038/s41467-020-20884-9)
Supplement: Supplementary file 3 — Description of Additional Supplementary Files [file 41467_2020_20884_MOESM3_ESM.pdf]

Description of Additional Supplementary Files

Supplementary Movie 1: The reconstructed Cryo-ET volumes of the polystyrene nanoparticles.

Supplementary Movie 2: The reconstructed Cryo-ET volumes of the biomolecular corona coated polystyrene nanoparticles after interactions with 10% human plasma.

Supplementary Movie 3: The reconstructed Cryo-ET volumes of the biomolecular corona coated polystyrene nanoparticles after interactions with 50% human plasma.

Supplementary Movie 4: The 3D reconstructed Cryo-ET volumes of the reconstructed biomolecular corona structure (blue and yellow colors show nanoparticles and protein clusters, respectively).

Supplementary Movies 5 to 23: Show a 3D reconstruction of the biomolecular corona around selected nanoparticles.
-The coordinates of the center of mass (as detected by the analysis code) of the NP is displayed at the beginning of the videos (z: photo stack number, x,y: pixel coordinates of the center of mass in the image).
-On the bottom left of the video the sample tag together with the concentration classification is displayed (LowCone and HighCone correspond to the 10% and 50% plasma concentrations).
-On the bottom right of the video the nanoparticle's number tag is used to distinguish between the nanoparticles in the 10% and 50% plasma concentrations.

Blue voxels (3d pixels) show the nanoparticles and the white voxels show protein clusters detected around the surface of nanoparticles by the analysis code.
