# Supplementary material for: Nanoscale characterization of the biomolecular corona by cryo-electron microscopy, cryo-electron tomography, and image simulation
Source: Nat Commun. 2021 Jan 25;12:573. doi: 10.1038/s41467-020-20884-9 (PMC7835367; doi:10.1038/s41467-020-20884-9)
Supplement: Supplementary file 27 — Reporting Summary [file 41467_2020_20884_MOESM27_ESM.pdf]

nature research

Corresponding author(s): DBPR NCOMMS-20-11969A-Z

Last updated by author(s): ~~September 11, 2020~~

Reporting Summary

Nature Research wishes to improve the reproducibility of the work that we publish. This form provides structure for consistency and transparency in reporting. For further information on Nature Research policies, see Authors & Referees and the Editorial Policy Checklist .

Statistics

For all statistical analyses, confirm that the following items are present in the figure legend, table legend, main text, or Methods section.

n/a	Confirmed	
☒	☐	The exact sample size (*n*) for each experimental group/condition, given as a discrete number and unit of measurement
☒	☐	A statement on whether measurements were taken from distinct samples or whether the same sample was measured repeatedly
☒	☐	The statistical test(s) used AND whether they are one- or two-sided<br>*Only common tests should be described solely by name; describe more complex techniques in the Methods section.*
☐	☒	A description of all covariates tested
☐	☒	A description of any assumptions or corrections, such as tests of normality and adjustment for multiple comparisons
☐	☒	A full description of the statistical parameters including central tendency (e.g. means) or other basic estimates (e.g. regression coefficient) AND variation (e.g. standard deviation) or associated estimates of uncertainty (e.g. confidence intervals)
☒	☐	For null hypothesis testing, the test statistic (e.g. *F*, *t*, *r*) with confidence intervals, effect sizes, degrees of freedom and *P* value noted<br>*Give P values as exact values whenever suitable.*
☒	☐	For Bayesian analysis, information on the choice of priors and Markov chain Monte Carlo settings
☒	☐	For hierarchical and complex designs, identification of the appropriate level for tests and full reporting of outcomes
☒	☐	Estimates of effect sizes (e.g. Cohen's *d*, Pearson's *r*), indicating how they were calculated

Our web collection on statistics for biologists contains articles on many of the points above.

Software and code

Policy information about availability of computer code

Data collection	1- Transmission Electron Microscopy (FEI Tecnai G2 F20 200 kV TEM equipped with a Gatan Ultrascan 4000 CCD) was used for negative staining imaging.<br>2- Titan Krios 300 kV Cryo-S/TEM equipped with a Falcon 2 direct electron detector (DED) (Thermo Fischer Scientific): was used for Cryo-EM image collection<br>3- Titan Krios TEM (Thermo Fischer Scientific) operated at 300kV and equipped with Falcon 2 Direct Detection Device (DDD) (Thermo Fischer Scientific) using FEI Batch Tomography Software: was used for Single Axis tomograms
Data analysis	1- All tomograms were aligned, filtered and reconstructed using IMOD.4.9.11. Back projection method and iterative reconstruction technique (SIRT) was used.<br>2- 3D visualization and volume were done using Chimera. The movies 1,2, and 3 were generated using the UCSF Chimera software version 1.5.1<br>3- The movies 4-32 were generated using ImageJ software version 2.0.0<br>4- Image processing and structure analysis utilised using C++ code publicly available at https://github.com/afarnudi/Corona_Structure_anlysis

For manuscripts utilizing custom algorithms or software that are central to the research but not yet described in published literature, software must be made available to editors/reviewers. We strongly encourage code deposition in a community repository (e.g. GitHub). See the Nature Research guidelines for submitting code & software for further information.

Data

Policy information about availability of data

All manuscripts must include a data availability statement. This statement should provide the following information, where applicable:

- Accession codes, unique identifiers, or web links for publicly available datasets
- A list of figures that have associated raw data
- A description of any restrictions on data availability

The authors declare that the data supporting the findings of this study are available within the paper and its supplementary information files.

Field-specific reporting

Please select the one below that is the best fit for your research. If you are not sure, read the appropriate sections before making your selection.

☒ Life sciences ☐ Behavioural & social sciences ☐ Ecological, evolutionary & environmental sciences

For a reference copy of the document with all sections, see nature.com/documents/nr-reporting-summary-flat.pdf

Life sciences study design

All studies must disclose on these points even when the disclosure is negative.

Sample size	N/a
Data exclusions	N/a
Replication	N/a
Randomization	N/a
Blinding	N/a

Reporting for specific materials, systems and methods

We require information from authors about some types of materials, experimental systems and methods used in many studies. Here, indicate whether each material, system or method listed is relevant to your study. If you are not sure if a list item applies to your research, read the appropriate section before selecting a response.

Materials & experimental systems

n/a	Involved in the study	
☒	☐	Antibodies
☒	☐	Eukaryotic cell lines
☒	☐	Palaeontology
☒	☐	Animals and other organisms
☒	☐	Human research participants
☒	☐	Clinical data

Methods

n/a	Involved in the study	
☒	☐	ChIP-seq
☒	☐	Flow cytometry
☒	☐	MRI-based neuroimaging
